# Length of paediatric inpatient stay, socio-economic status and hospital configuration: a retrospective cohort study

**DOI:** 10.1186/s12913-017-2171-x

**Published:** 2017-04-17

**Authors:** Michelle Heys, Matthew Rajan, Mitch Blair

**Affiliations:** 1 Child Public Health Group, Imperial College River Island Academic Centre, Paediatric Department, Northwick Park Hospital (NWLH NHS Trust), London, UK; 2 Institute for Global Health, University College London, 30 Guildford Street, London, WC1N 1EH UK; 3 Imperial College London, London, UK; 4 Great Ormond Street Institute of Child Health, University College London, 30 Guildford Street, London, WC1N 1EH UK

**Keywords:** Length of stay, Socioeconomic status, Health services research, Paediatrics, British health service, National

## Abstract

**Background:**

Variation in paediatric inpatient length of stay exists – whether this is driven by differences in patient characteristics or health service delivery is unclear. We will test the hypotheses that higher levels of deprivation are associated with prolonged length of stay and that differences in prolonged length of stay across 2 hospitals will be explained by demographic, clinical and process factors.

**Methods:**

This is a retrospective cohort study of 2889 children aged less than 16 years admitted from 1st April 2009 to 30^th^ March 2010. Administrative data were used from two UK hospitals whose Accident and Emergency (A&E) departments were paediatric and adult physician led respectively. The main outcome was prolonged length of stay defined as greater than or equal to the mean (1.8 days). Sensitivity analyses defined prolonged length of stay as greater than the median (1 day). Demographic, clinical and process characteristics were examined. Socio-economic position was measured by Income Deprivation Affecting Children Index. Multivariable logistic and linear regression analyses were performed.

**Results:**

We did not find a consistent association between length of stay and socio-economic position, using a variety of definitions of length of stay. In contrast, adjusted for age, gender, socio-economic position, ethnicity, final diagnosis, number of hospital admissions, source of admission, and timing of admission, admission to the adult led A&E hospital was more strongly associated with prolonged length of stay (Odds Ratio 1.41, 95% Confidence Interval 1.16, 1.71).

**Conclusion:**

Local variation in paediatric inpatient length of stay was not explained by demographic, clinical or process factors, but could have been due to residual confounding by medical complexity. Length of stay was not consistently associated with socio-economic position suggesting that length of stay is a function of health service not the determinants of health. Analyses of these types of data would be strengthened by measures of complexity and adverse events.

## Background

Across the United Kingdom (UK) emergency admission rates and short stay admissions for minor illness in children are rising [1, 2]. In comparison mean inpatient length of stay (LOS) in the UK has fallen in the last 30 years from 3.9 to 1.9 days [3, 4]. A significant variation in LOS exists with mean LOS reported in 2009 of between 0.1 and 4.4 days for children and young people across the UK [5]. Research into which factors contribute to this variation is urgently required, especially in children. Inpatient LOS is used as a measure of quality of health service [6]. Adverse clinical events are associated with prolonged LOS, [7, 8] and there is evidence to suggest that shorter length of stay does not compromise care quality [9]. Few studies have examined factors associated with LOS for acute paediatrics and these have mostly looked at the association with socio-economic position (SEP) [10, 11]. These have been inconclusive possibly due to differences in definition of LOS, age, diagnostic groups studied and measure of SEP used. Taking a single well defined illness such as bronchiolitis in under twos, an exploration of UK wide Hospital Episode Statistics (HES) found 6 fold variation in mean LOS by primary care trust (PCT), however they did not find LOS to be associated with SEP (Index of Multiple Deprivation (IMD) by PCT, 2010) [10]. In contrast, a similar study of UK HES found that lower SEP using two PCT level measures (IMD and selected domains of Child and Well Being Indices) was associated with LOS greater than 4 or more days for children under 14 years admitted for breathing difficulty, feverish illness and diarrhoea [11] LOS less than 24 h, was associated with a diagnosis of ingestion, night time admission and admission via the Accident and Emergency Department (A&E) but not SEP (Carstairs Index by individual postcode) [12]. Cumulative LOS from birth to 10 years was associated with father’s occupation at birth and this association was strongest between the ages of 3 and 10 years [13]. In a study of US teaching hospitals, female sex, black race, age < 30 days, greater illness severity and complexity were associated with prolonged LOS [14]. Finally admission to a US teaching hospital was associated with prolonged LOS which was not entirely explained by chronicity of clinical diagnosis, ethnicity, age or gender [15]. Thus both service configuration and patient SEP may be important factors in determining LOS.

This study adds to the limited published evidence in this area by examining the association between clinical and demographic characteristics of patients and hospital and service characteristics with LOS for acute inpatient paediatrics using individual level data for inpatients for all measures except SEP which is measured at lower super output area level (LSOA). Previous studies have used aggregated measures and limited analyses by disease category or age group. We additionally seek to explore the relation between hospital A&E configuration and LOS. We will test the hypothesis that higher levels of deprivation are associated with prolonged LOS and that differences in LOS by hospital site, if present, will be in part explained by demographic, clinical and process factors.

## Methods

### Study design

This is a retrospective cohort study of 2889 children and young people aged 16 years and under.

### Study population

Only those who were admitted to the inpatient paediatric wards were included, thus patients admitted to short stay or ambulatory wards were excluded. Of the 14,416 inpatients aged 16 years or below, who were admitted during the study year, 5343 were admitted to postnatal and neonatal wards, 3132 to day care units, 724 to the day surgery unit, 2138 to the paediatric A&E observation bay and 189 to other hospital wards. These data were excluded. An additional 67 were excluded due to being significant geographical outliers (distance lived from hospital greater than 25 km). This left the study population of 949 and 1940 children and young people (2889 in total) who were admitted electively or as an emergency to the inpatient paediatric wards of Hospitals P and A respectively from 1st April 2009 to 30^th^ March 2010.

### Setting

Two hospitals which are part of a single National Health Service (NHS) Hospital Trust in London, UK (serving a population of around 500,000 people in total), with different models of Paediatric emergency care; Hospital P A&E was paediatric led with all children aged less than 16 years being seen by a paediatrican, Hospital A was led by adult emergency physicians with children aged less than 16 years only being seen by a paediatrician if an in-patient admission was likely. During the study period both hospitals had a functioning short stay observation (Hospital A) or ambulatory care unit (Hospital P).

Ethical approval was sought from the Ethics committee of the NHS Trust covering both Hospitals; however, they considered this a service review and thus exempt from formal ethics review. Permission to access the data was given by North West London NHS trust Research and Design Services.

### Data

All data were anonymised by a single researcher. Routinely collected hospital data (administrative data) were reviewed for all children recorded as having an inpatient admission during the study period.

### Outcome

**Fig. 1:**
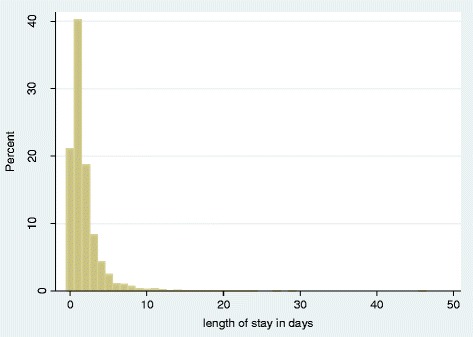
Percentage of children per length of stay (measured in complete days)

LOS was recorded in complete days. Zero LOS equated to an admission of less than 24 h duration. The main outcome of interest was prolonged LOS defined as greater than the mean LOS (1.8 days). Mean rather than median LOS was used for the main outcome as despite LOS not being normally distributed (Fig. 1), as this is the outcome most frequently reported in health service policy reports and in the literature. For completeness, all analyses were repeated using three other definitions and measures of length of stay additionally quoted in the literature namely: (1) LOS greater than the median of 24 h, [12] (2) LOS of 4 or more days [11] and finally (3) LOS as a continuous outcome in days [13].

### Exposures

Data were available on demographic, clinical and hospital process characteristics where process characteristics refers to factors such as source of referral to hospital and time of admission. Demographic characteristics included age (in years), sex, ethnicity, SEP and distance lived from hospital (categorised as per Table 1). Clinical factors included final diagnosis and readmission within 28 days.

**Table 1:** Subject characteristics by Hospital

Characteristics			Hospital A (*n* = 1940)^a^	Hospital P (*n* = 949)^a^	Overall (*n* = 2889)^a^	*p* value^b^
LOS	LOS (days)	Mean, SD	1.8 ± 2.4	1.7 ± 2.8	1.8 ± 2.6	0.654
	LOS ≥ 1.8 days		39.7	36.5	38.7	0.089
	LOS < 24 h		20	23.5	21.1	0.288
Demographic	Gender	Male	56.3	55.1	55.9	0.532
	Age (years)	Mean, SD	3.9 ± 4.4	3.8 ± 4.2	3.7 ± 4.2	0.937
	Age group (years)	1 and Under	40	40.9	40.3	0.509
		2 to 5	34.1	34.7	34.3	
		6 to 10	12.9	13.4	13.1	
		11 to 16	13	11.1	12.4	
	Ethnic Group	White	28.9	39.2	32.3	<0.001
		Mixed White	23.2	24	23.5	
		Asian	33.6	23.9	30.4	
		Black	8.4	5.2	7.3	
		Other or not specified	5.9	5.9	6.5	
	Distance (Km)^a^	Mean	6.3 ± 14.7	4.8 ± 14.7	6.3 ± 23.2	
	Distance (quartiles) ^a^	1st (live closest)	20	35	24.9	<0.001
		2nd	25	25.2	25.1	
		3rd	25.7	23.5	25	
		4th (live furthest)	29.2	16.3	25	
	IDACI	Mean	0.3 ± 0.14	0.43 ± 0.17	0.34 ± 0.17	
	IDACI (quartiles)^c^	1st (least deprived)	30.8	12.9	24.9	<0.001
		2nd	28.8	17.3	25	
		3rd	25.9	23	24.9	
		4th (most deprived)	14.6	46.8	25.1	
Clinical	Specialty	Medical	82.8	93.5	86.3	<0.001
		Surgical	17.2	6.5	13.7	
	Diagnosis	allergy	0.4	0.6	0.5	<0.001
		cardiac	1	0.7	0.9	
		endocrine	1.1	2.1	1.5	
		gastro	13.6	10.6	12.6	
		haemoncology	5.4	4.7	5.2	
		infection	21.6	21.6	21.6	
		MSK	1.5	1.7	1.6	
		neurology	6.1	9.3	7.1	
		other	2.4	1.9	2.3	
		psychosocial	1.3	1.8	1.5	
		respiratory	20	24.7	21.5	
		surgical GIT	1.8	1	1.5	
		trauma	12.7	12.1	13.2	
		urogenital	4.9	3.6	1.8	
		Maxfax/Dental/ ENT	3.9	1.4	3.1	
		congenital	0.4	0	0.3	
		Dermatology	2	2.2	2.1	
	Number of admissions (in the year)	1	64.5	71	66.6	0.005
		2	18.8	15.9	17.8	
		3	6.4	5.5	6.1	
		4 or more	10.4	7.6	9.5	
	Readmission within 28 days		2.5	3.4	2.8	0.196
Process	Time of admission	9.00 to 16.59	33.8	36.4	34.6	0.105
		17.00 to 21.59	27.5	28.9	28	
		22.00 to 08.59	38.7	34.7	37.4	
	Day of admission	weekday	85.7	88	86.5	0.095
		weekend	14.3	12	13.5	
	Season of admission	spring	26.8	28.1	27.2	0.58
		summer	23.8	23.8	23.8	
		autumn	25	25.8	25.2	
		winter	24.5	22.2	23.8	
	Source of admission	elective	9.6	3.6	7.7	<0.001
		emergency via A&E	74.6	88.6	79.2	
		emergency via other	13	6.9	11	
		other	27	1	2.1	

*A&E* Accident and Emergency department, *ENT* Ears nose and throat, *GIT* Gastrointestinal Tract, *IDACI* Income deprivation affecting children index, *LOS* Length of stay, *MaxFax* Maxillofacial, *MSK* Musculoskeletal, *SD* Standard deviation

^a^Data were complete for all variables except for distance lived from hospital (*n* = 74) due to missing postcode or mismatched hospital ward to hospital; % of children in each category unless indicated

^b^Univariate analysis of continuous variables using *t*-test (parametric) or Mann–Whitney (non-parametric) and of categorical variables using chi^2^

^c^Quartile of IDACI was calculated in absolute terms for the total dataset not by hospital site

SEP was measured using the Income Deprivation Affecting Children Index (IDACI) derived from the postcode. The IDACI gives both a rank and score for the lower super output area (LSOA) within which the postcode lies. The IDACI score ranges from 0 (lowest deprivation) to 1 (highest deprivation), such that a score of 0.24 would equate to 24% of children less than 16 years in that area living in families that are income deprived. Income deprived is defined as being in receipt of income support and an equivalised income which, excluding housing benefits and before housing costs, is less than 60% of the national median income. This 60% weekly threshold depends on household composition and varies from 119 to 288 UKP (2008/9 figures). The IDACI also ranks each LSOA from most to least deprived (range 0 – lowest deprived ranking - to 32,482 – most deprived ranking). Thus SEP is an area level not an individual level measure.

The distance lived from hospital to home was calculated using the Department for Education, UK online tool which calculates the straight line distance between two postcodes [16]. The clinical characteristics assessed were final diagnosis and admission under surgical versus non-surgical consultant team. Case notes are routinely reviewed by diagnostic coding staff and a diagnosis and diagnostic category entered into the hospital activity database. These categories are taken from the NHS National Data Dictionary- a standard which is used in all hospitals [17] and it was these categories that were taken as the final diagnostic category for each patient. Surgical versus non-surgical admission team was determined by the clinical specialty of the admitting consultant. Hospital process characteristics included: hospital site, date, day, season and time of admission, number of hospital admissions during the study period and source of admission (elective, emergency via A&E, emergency via other source such as General Practitioner and other (included transfers from other hospitals).

### Statistical methods

Distribution of LOS as a continuous variable in days was assessed. Multivariable logistic regression was used to assess potential independent associations for the binary outcomes for definitions of prolonged length of stay as follows: (1) greater than the mean (1.8 days), (2) greater than the median (24 h) and (3) greater than or equal to 4 days [11]. Negative binomial regression analyses reporting Incident Rate Ratios (IRR) was used to assess potential independent associations for length of stay as a continuous outcome in days.. We present 4 models; model 1 adjusted for IDACI quartile and admission hospital only; model 2 additionally adjusted for other available demographic factors, namely: age, gender, and ethnicity; model 3 additionally adjusted for clinical factors, namely: final diagnosis, medical or surgical specialty and number of hospital admissions during the study period; and model 4 additionally adjusted for other factors, namely: source of admission, weekend or weekday admission, season of admission, time of admission and readmission within 28 days. This model strategy was chosen to assess in a step-wise manner the impact of demographic, clinical and then process factors on the relation between admission hospital and IDACI quartile and LOS.

IDACI and distance lived from hospital were analysed in 2 ways: (1) continuous variable and (2) absolute quartiles in order to assess whether there was a linear or non-linear relation between income deprivation and distance lived from hospital site and LOS. A linear relation assumes that two quantities are proportional to each other (ie. doubling of one results in doubling of the other). A non-linear relation is not based on this assumption.

Sensitivity analysis included repetition of all analyses restricting the dataset in 3 ways, firstly to children with LOS less than 10 days to check for influence of significant outliers (48 children had a LOS greater than 10 days), secondly to those aged less than 2 years with bronchiolitis to compare our findings with those of Cheung et al. [10] and third to those children who experienced one admission only, to check for effects of clustering of risk factors in children with repeat admissions.

Data analysis was conducted using STATA v12 (STATA Corporation, College Station, Texas) and R 3.0.2 [18].

## Results

LOS, recorded in days, ranged from 0 to 46 (median 1 (interquartile range (IQR) 1–2), mean 1.8 (standard deviation (SD) 2.5, variance 6.6)). A fifth of children (21.1%) were admitted for less than 24 h (0 days) and 35% experienced more than one admission per year. A minority (2.8%) of clinical episodes were readmissions within 28 days. Length of stay in days was positively skewed and showed evidence of over dispersion (variance greater than SD) (Fig. 1).

Table 1 shows demographic, clinical and process characteristics by hospital. Children admitted to Hospital P (with a paediatric led A&E) were more likely: to be of Asian or Black ethnicity, live further away from the hospital, come from lower SEP, to have an endocrinological or neurological diagnosis, to have had fewer admissions over the year and to be admitted electively.

Table 2 shows characteristics by prolonged LOS (greater than the mean, 1.8 days). On univariate analysis, prolonged LOS was associated with younger age, having had more admissions over the year and being admitted under a medical team and via A&E.

**Table 2:** Subject characteristics by prolonged length of stay defined as greater than or equal to the mean of 1.8 days

Characteristic			prolonged length of stay		Overall (*n* = 2889)^a^	*p* value^b^
			Less than 1.8 days (*n* = 1772) ^a^	Greater than or equal to 1.8 days (*n* = 1117)^a^		
Hospital site	P (Paediatric led A&E)		34	31	61.3	0.089
	A (Adult physician led A&E)		66	69		
Demographic	Gender	Male	56.4	55.2	55.9	0.584
		Female	43.6	44.8		
	Age (years)	mean, SD	4.2 ± 4.4	3.4 ± 4.4	3.9 ± 4.4	<0.001
	Age group (years)	1 and Under	35.4	48	40.3	<0.001
		2 to 5	36.9	30.1	34.3	
		6 to 10	14.2	11.3	13.1	
		11 to 16	13.5	10.7	12.4	
	Ethnic Group	White	32.3	32.2	32.3	0.706
		Mixed White	23.3	23.8	23.5	
		Asian	30.5	3.3	30.4	
		Black	7	7.9	7.3	
		Other/unspecified	6.9	5.8	6.5	
	Distance (Km)^a^	Mean	6.5 ± 24.8	6.1 ± 20.3	6.3 ± 23.2	0.107
	Distance (quartiles)^a^	1st (closest)	23.5	27.2	24.9	0.135
		2nd	25.8	24	25.1	
		3rd	25	25.1	25	
		4th (furthest)	25.8	23.8	25	
	IDACI	Mean	0.3 ± 0.14	0.43 ± 0.17	0.34 ± 0.17	0.685
		Median	0.31	0.32	0.32	
	IDACI (quartiles)^c^	1st (least deprived)	25.3	24.3	24.9	<0.001
		2nd	25.1	24.8	25	
		3rd	23.3	27.6	24.9	
		4th (most deprived)	26.3	23.3	25.1	
Clinical	Specialty	Medical	82.1	93	86.3	<0.001
		Surgical	18	6.9	13.7	
	Diagnosis	allergy	0.5	0.5	0.5	<0.001
		cardiac	0.8	1.1	1.5	
		endocrine	1.2	1.9	2.6	
		gastro	12.7	12.5	12.6	
		haemoncology	4.8	5.7	5.2	
		infection	20.2	23.8	21.6	
		MSK	1.8	1.2	1.6	
		neurology	7.6	6.5	7.1	
		other	2.3	2.2	2.3	
		psychosocial	1.9	9	1.5	
		respiratory	19.6	24.5	21.5	
		surgical GIT	6.2	2.9	1.5	
		trauma	16.5	6.2	12.5	
		urogenital	3.8	5.4	4.4	
		Maxfax/Dental/ENT	3.9	1.8	3.1	
		congenital	0.3	0.3	0.3	
		Dermatology	1.7	2.7	2.1	
	Number of admissions in the year	1	68.2	64.2	66.6	0.038
		2	17.7	18.1	17.8	
		3	5.3	7.4	6.1	
		4 or more	8.9	10.3	9.5	
	Readmission within 28 days		2.8	2.8	2.8	0.941
Process	Time of admission	9.00 to 16.59	33.5	36.4	34.6	<0.001
		17.00 to 21.59	24	34.4	28	
		22.00 to 08.59	42.6	29.2	37.4	
	Day of admission	weekday	85.9	87.4	86.5	0.256
		weekend	14.1	12.6	13.5	
	Season of admission	spring	26.4	28.6	27.2	0.58
		summer	24.6	22.6	23.8	
		autumn	24.7	26.1	25.2	
		winter	24.4	22.7	23.8	
	Source of admission	elective	11	2.4	7.7	<0.001
		emergency via A&E	75.6	85.1	79.2	
		emergency via other	12.3	9	11	
		other	11.9	3.6	2.1	

*A&E* Accident and Emergency department, *ENT* Ears nose and throat, *GIT* Gastrointestinal Tract, *IDACI* Income deprivation affecting children index, *LOS* Length of stay, *MaxFax* Maxillofacial, *MSK* Musculoskeletal, *SD* Standard deviation

^a^Data were complete for all variables except for distance lived from hospital (*n* = 74) due to missing postcode or mismatched hospital ward to hospital

^b^Univariate analysis of continuous variables using *t*-test (parametric) or Mann–Whitney (non-parametric) and of categorical variables using chi^2^

^c^Quartile of IDACI was calculated in absolute terms for the total dataset not by hospital

% of children in each category unless indicated

Table 3 shows odds of prolonged LOS, defined in 4 ways. Adjusted for admission hospital (Model 1), being in the 3^rd^ (more deprived) IDACI quartile was associated with increased odds of prolonged LOS above the mean of 1.8 days (Odds Ratio (OR) 1.26, 95% Confidence interval (CI) 1.02, 1.56). Similarly adjusted, being in the 3^rd^ (more deprived) IDACI quartile was associated with increased length of stay as a continuous variable in days (IRR 1.16 CI (1.04, 1.30). These associations were attenuated by additional adjustment for other demographic, clinical and process factors. (Table 3: Models 2 to 4). There was no significant association between IDACI and LOS longer than the two other definitions of prolonged LOS of median of 24 h or 4 days.

**Table 3:** Association of Socio-economic position (SEP) with Prolonged length of stay (LOS)

		Model 1			Model 2			Model 3			Model 4		
LOS	IDACI (quartiles)	OR	95% CI	*p* value	OR	95% CI	*p* value	OR	95% CI	*p* value	OR	95% CI	*p* value
≥ to 1.8 days (mean)	1st (least)	1.00	-	-	1.00	-	-	1.00	-	-	1.00	-	-
	2nd	1.04	0.84, 1.29	0.696	1.07	0.86, 1.34	0.54	1.07	0.85, 1.35	0.538	1.03	0.81, 1.30	0.824
	3rd	1.26	1.02, 1.56	0.035	1.29	1.04, 1.61	0.023	1.27	1.01, 1.59	0.041	1.24	0.98, 1.57	0.068
	4th (most)	0.98	0.78, 1.24	0.757	0.97	0.77, 1.23	0.795	1.00	0.78, 1.27	0.992	0.96	0.75, 1.23	0.746
≥to 4 days	1st (least)	1.00	-	-	1.00	-	-	1.00	-	-	1.00	-	-
	2nd	1.13	0.88, 1.49	0.325	1.16	0.88, 1.52	0.285	1.18	0.89, 1.56	0.246	1.17	0.88, 1.57	0.288
	3rd	1.27	0.97, 1.64	0.069	1.28	0.98, 1.68	0.072	1.23	0.93, 1.62	0.151	1.20	0.91, 1.59	0.196
	4th (most)	0.96	0.70, 1.25	0.767	0.93	0.69, 1.25	0.638	0.96	0.71, 1.30	0.813	0.96	0.71, 1.30	0.789
>24 h (median)	1st (least)	1.00	-	-	1.00	-	-	1.00	-	-	1.00	-	-
	2nd	1.21	0.94, 1.56	0.1516	1.19	0.92, 1.55	0.189	1.17	0.90, 1.54	0.237	1.05	0.79, 1.41	0.730
	3rd	1.17	0.91, 1.51	0.221	1.17	0.90, 1.52	0.233	1.14	0.87, 1.49	0.359	1.11	0.83, 1.49	0.476
	4th (most)	1.18	0.90, 1.54	0.230	1.14	0.87, 1.50	0.33	1.18	0.89, 1.57	0.249	1.07	0.79, 1.45	0.670
		IRR	95% CI	*p* value	IRR	95% CI	*p* value	IRR	95% CI	*p* value	IRR	95% CI	*p* value
Days	1st (least)	ref	-	-	ref	-	-	ref	-	-	ref	-	-
	2nd	1.07	0.95, 1.19	0.298	1.06	0.95, 1.19	0.310	1.06	0.94, 1.18	0.351	1.05	0.93, 1.17	0.443
	3rd	1.16	1.04, 1.30	0.009	1.17	1.04, 1.31	0.009	1.14	1.02, 1.28	0.022	1.13	1.01, 1.27	0.027
	4th (most)	1.14	1.01, 1.29	0.031	1.12	0.99, 1.27	0.061	1.13	1.01, 1.28	0.040	1.12	1.00, 1.26	0.056

*CI* Confidence interval, *IDACI* Income deprivation affecting children index, *IRR* Incident Rate Ratio, *LOS* Length of stay, *OR* Odds ratio; ref: reference category

Model 1: Adjusted for admission hospital and quartile of IDACI; as defined in Tables 1 and 2

Model 2: Additionally adjusted for age (groups), gender, ethnicity (group) - as defined in Tables 1 and 2.

Model 3: Additionally adjusted for final diagnosis, medical or surgical specialty and number of hospital admissions during the study period

Model 4: Additionally adjusted for source of admission, weekend or week day admission, season and time of admission and readmission within 28 days- as defined in Tables 1 and 2)

Table 4 shows odds and risk of prolonged LOS and hospital of admission. Admission to Hospital A (adult led A&E) was associated with increased odds and risk of prolonged LOS using every definition, except for prolonged LOS of 4 or more days. Adjusting for demographic, clinical and process factors strengthened the association between admission to Hospital A and prolonged LOS greater than the mean of 1.8 days (Table 4, Model 4: OR 1.41, 95% CI 1.16, 1.71).

**Table 4:** Association of Hospital of admission (Hospital P: A&E paediatric led; Hospital A: Adult A&E led) with Prolonged length of stay (LOS)

		Model 1			Model 2			Model 3			Model 4		
LOS	Hospital	OR	95% CI	*p* value	OR	95% CI	*p* value	OR	95% CI	*p* value	OR	95% CI	*p* value
≥1.8 days (mean)	P	1.00	-	-	1.00	-	-	1.00	-	-	1.00	-	-
	A	1.15	0.97, 1.37	0.104	1.22	1.02, 1.46	0.030	1.31	1.09, 1.58	0.004	1.41	1.16, 1.70	<0.001
≥4 days	P	1.00	-	-	1.00	-	-	1.00	-	-	1.00	-	-
	A	1.06	0.85, 1.31	0.614	1.09	0.87, 1.35	0.456	1.12	0.89, 1.41	0.323	1.14	0.91, 1.44	0.262
>24 h (median)	P	1.00	-	-	1.00	-	-	1.00	-	-	1.00	-	-
	A	1.28	1.05, 1.57	0.015	1.38	1.12, 1.69	0.002	1.65	1.32, 2.05	<0.001	1.95	1.53, 2.48	<0.001
		IRR	95% CI	*p* value	IRR	95% CI	*p* value	IRR	95% CI	*p* value	IRR	95% CI	*p* value
Days	P	ref	-	-	ref	-	-	ref	-	-	ref	-	-
	A	1.06	0.97, 1.16	0.194	1.09	0.99, 1.19	0.075	1.14	1.04, 1.26	0.005	1.13	1.04, 1.23	0.003

*CI* Confidence interval, *A&E* Accident and Emergency department, *IDACI* Income deprivation affecting children index, *IRR* Incident Rate Ratio, *LOS* Length of stay, *OR* Odds ratio, *ref* reference category

Model 1: Adjusted for admission hospital and quartile of IDACI; as defined in Tables 1 and 2

Model 2: Additionally adjusted for age (groups), gender, ethnicity (group) - as defined in Tables 1 and 2

Model 3: Additionally adjusted for final diagnosis, medical or surgical specialty and number of hospital admissions during the study period

Model 4: Additionally adjusted for source of admission, weekend or week day admission, season and time of admission and readmission within 28 days - as defined in Tables 1 and 2)

### Sensitivity analyses

There was no association between SEP and any definition of LOS when these analyses were restricted to those aged under 2 years (as per Cheung et al. [10]) – however associations between hospital of admission and prolonged LOS were consistent and similar to analyses of all ages (data not shown). Restriction of analyses to those with length of stay less than 10 days did not alter the associations between SEP and hospital of admission or prolonged LOS as greater than the mean (1.8 days), the median (24 h) or 4 days (data not shown). Restriction of analyses to only one admission in order to check for clustering of characteristics for repeat attenders attenuated findings. Of note the association between being in the 3^rd^ (more deprived) IDACI quartile and prolonged length of stay was no longer statistically significant. (Table 5, Appendix) The association between hospital site of admission and prolonged length of stay remained statistically significant (Table 6, Appendix).

Table 7 (Appendix) shows fully adjusted associations (as per Model 4 from Table 3) between all clinical, demographic and process factors (except for SEP, hospital site, gender and ethnicity) for each definition of prolonged LOS. The only consistent associations across the definitions of prolonged LOS were that being older than 1 year of age was associated with reduced odds and risk of prolonged LOS, and emergency admission via A&E or transfer from another hospital was associated with increased odds and risk of prolonged LOS. Neither gender nor ethnicity were associated with any measure of inpatient LOS. The following factors were associated with increased odds of LOS greater than the mean of 1.8 days: being admitted 3 times in the study period, final diagnosis of a surgical gastrointestinal (GIT) disorder and emergency source of admission (via A&E, via other sources (including General practitioner (GP) referrals) and other (including transfers back from other hospitals and intensive care units. In post hoc analysis, surgical gastro-intestinal diagnoses were reviewed in more detail. Mean length of inpatient stay was more than 1.8 days for a diagnosis of appendicitis (simple and complicated) and for those admitted and diagnosed with a colostomy/enterostomy malfunction.

## Discussion

The hospital site of admission was more strongly and consistently associated with prolonged LOS than was a measure of SEP. Patients admitted to Hospital P with a paediatric led A&E service had 50% lower odds of experiencing a prolonged LOS than those admitted to Hospital A. However, we were not able to ascertain from these data whether this association could be attributed to differences in service configuration and/or of quality of care. We did not find a convincing/significant association between LOS and SEP using a variety of definitions of LOS. We observed a nonlinear association between prolonged LOS and SEP that was attenuated to non-significance after adjustment for other factors. The third most deprived quartile of deprivation was associated with increased odds of LOS greater than 1.8 days. Our findings of a weak and inconsistent association between SEP and LOS are largely consistent with findings in other settings and patient groups [10, 11]. To our knowledge, the possibility of a nonlinear relation between SEP and LOS has not been examined previously. However, a similar nonlinear association has been shown in the relation between prevalence of obesity and SEP [19].

It may be that once other clinical, demographic and hospital process characteristics are taken into account a child’s SEP has minimal influence on their LOS for a given admission. Although this seems counterintuitive recent evidence at the population level shows that worse health outcomes for children at the population level may be independent of the levels of deprivation [20]. However, it is unclear why odds of prolonged LOS would be similar for those in the least and most deprived quartiles for IDACI. It could be that this is a chance finding or it may be due to the fact that once admitted, children receive equal care regardless of SEP and so are discharged in a similar timeframe. The IDACI used is based on income; other measures of SEP may have different relations with LOS.

The observed relation between hospital and prolonged LOS could have been due to chance or residual confounding. However, the fact that the association strengthened when all available factors were taken into account suggests this may be a significant finding. It is not possible to comment from these data as to whether this represents better or worse quality of care. To our knowledge there were not specific differences in clinical pathways as clinical guidelines and protocols were kept on a shared computer drive and it is unlikely that these will have differed significantly between the sites, although differential adherence to these cannot be discounted and factors such as these were not measured in a systematic way in this study. Of note, shorter LOS in Hospital P did not appear to be associated with repeat admissions as children in Hospital P had fewer total annual admissions than those in Hospital A (Table 1). Morse et al. demonstrated that reduced inpatient LOS did not lead to more frequent admissions across 43 hospitals in the United States [9]. It is possible that patients admitted to Hospital A had greater medical complexity or illness severity, both of which have been associated with longer LOS [7, 8, 21]. We attempted to take this into account by adjusting for final diagnosis, but could not account for co-morbidity. Alternatively, Hospital A may have had a higher rate of adverse events, which have also been associated with prolonged LOS [7, 8].

Limitations of this study are that it is observational and therefore can only show association, not causation. Analysis is also based on routinely collected information and could be strengthened by key measures of illness severity (e.g. the Paediatric Early Warning Score (PEWS)), [22] medical complexity, co-morbidity, adverse events (e.g. Paediatric Trigger Tool) [23] and more detailed and varied measures of hospital process characteristics. We report differences between two hospitals only gathering individual level data across a number of institutions is difficult unless aggregated measures only are analysed losing a wealth of detail that this study measures.

Strengths of this study include its use of individual level data, relatively large sample size, the inclusion of all possible clinical conditions and paediatric age groups and the comparison between two hospitals. To our knowledge no study has examined detailed factors associated with local variation in LOS in a UK setting. Strengths of our study are that we use routinely collected data to explore a key quality outcome for inpatient care. Furthermore, we examined a range of definitions of prolonged LOS – including mean and median measures - and tested each of our main exposures against them. In addition, we tested for a nonlinear relation between SEP and LOS. There is a wealth of local and national data with which to conduct health services research within the UK [24]. Health services research is a growing field internationally and this paper demonstrates both the advantages and limitations of using routine hospital data to tackle key public health and health service issues.

## Conclusion

In this setting, hospital site of admission was clinically and statistically significantly associated with LOS and contrary to expectation, this finding was strengthened by consideration of all routinely recorded clinical, demographic and hospital process factors. These findings show that significant variation in LOS exists even at a local level; however quality of care cannot be assessed on LOS alone. Associations between SEP and prolonged LOS were less clear. Routine hospital data should include measures of medical complexity, illness severity and adverse events to facilitate more thorough exploration of variation in health care.

## Appendix

**Table 5:** Association of Socio-economic position (SEP) with Prolonged length of stay (LOS) for children with only one admission in the year of study (n=1925)

		Model 1			Model 2			Model 3			Model 4		
LOS	IDACI (quartiles)	OR	95% CI	p value	OR	95% CI	p value	OR	95% CI	p value	OR	95% CI	p value
≥ to 1.8 days (mean)	1st (least)	1.00	-	-	1.00	-	-	1.00	-	-	1.00	-	-
	2nd	1.01	0.78, 1.32	0.941	1.00	0.76, 1.31	0.999	0.97	0.73, 1.29	0.842	1.03	0.81, 1.30	0.824
	3rd	1.23	0.95, 1.61	0.120	1.21	0.92, 1.59	0.170	1.17	0.88, 1.55	0.288	1.24	0.98, 1.57	0.068
	4th (most)	0.88	0.67, 1.16	0.357	0.84	0.63, 1.12	0.244	0.86	0.64, 1.16	0.325	0.96	0.75, 1.23	0.746
≥to 4 days	1st (least)	1.00	-	-	1.00	-	-	1.00	-	-	1.00	-	-
	2nd	1.32	0.94, 1.84	0.107	1.28	0.91, 1.81	0.159	1.24	0.87, 1.76	0.240	1.23	0.89, 1.75	0.262
	3rd	1.21	0.86, 1.70	0.280	1.15	0.81, 1.64	0.438	1.12	0.78, 1.60	0.549	1.11	0.77, 1.60	0.576
	4th (most)	0.89	0.62, 1.28	0.520	0.81	0.55, 1.18	0.277	0.82	0.56, 1.20	0.307	0.84	0.57, 1.23	0.376
>24 hours (median)	1st (least)	1.00	-	-	1.00	-	-	1.00	-	-	1.00	-	-
	2nd	1.13	0.82, 1.57	0.446	1.08	0.77, 1.50	0.665	1.08	0.76, 1.52	0.675	1.04	0.72, 1.52	0.821
	3rd	0.98	0.71, 1.35	0.8961	0.93	0.67, 1.29	0.650	0.87	0.62, 1.22	0.421	0.90	0.62, 1.31	0.584
	4th (most)	0.91	0.66, 1.26	0.582	0.88	0.63, 1.22	0.433	0.93	0.66, 1.30	0.664	0.96	0.66, 1.39	0.545
		IRR	95% CI	p value	IRR	95% CI	p value	IRR	95% CI	p value	IRR	95% CI	p value
Days	1st (least)	ref	-	-	ref	-	-	ref	-	-	ref	-	-
	2nd	1.13	0.95, 1.19	0.069	1.09	0.96, 1.25	0.191	1.07	0.94, 1.22	0.327	1.06	0.93, 1.21	0.421
	3rd	**1.17**	**1.02, 1.34**	**0.021**	1.13	0.99, 1.30	0.070	1.12	0.98, 1.28	0.098	1.11	0.97, 1.27	0.119
	4th (most)	1.01	0.83, 1.16	0.894	0.95	0.83, 1.10	0.511	0.97	0.84, 1.11	0.622	0.98	0.85, 1.12	0.7595

**Table 6:** Association of Hospital of admission (Hospital P: A&E paediatric led; Hospital A: Adult A&E led) with Prolonged length of stay (LOS) for children with only one admission in the year of study (n=1925)

		Model 1			Model 2			Model 3			Model 4		
LOS	Hospital	OR	95% CI	p value	OR	95% CI	p value	OR	95% CI	p value	OR	95% CI	p value
≥1.8 days (mean)	P	1.00	-	-	1.00	-	-	1.00	-	-	1.00	-	-
	A	1.15	0.93, 1.41	0.200	1.22	0.98, 1.52	0.071	1.35	1.07, 1.69	0.010	1.44	1.14, 1.82	0.002
≥4 days	P	1.00	-	-	1.00	-	-	1.00	-	-	1.00	-	-
	A	0.93	0.71, 1.21	0.591	0.95	0.72, 1.25	0.711	1.00	0.76, 1.33	0.978	1.06	0.79, 1.41	0.707
>24 hours (median)	P	1.00	-	-	1.00	-	-	1.00	-	-	1.00	-	-
	A	1.15	0.93, 1.41	0.200	1.26	0.99, 1.61	0.059	1.40	1.09, 1.79	0.009	1.75	1.14, 1.82	<0.001
		IRR	95% CI	p value	IRR	95% CI	p value	IRR	95% CI	p value	IRR	95% CI	p value
Days	P	ref	-	-	ref	-	-	ref	-	-	ref	-	-
	A	1.07	0.96, 1.19	0.212	1.10	0.99, 1.23	0.067	1.19	1.07, 1.33	0.001	1.20	1.08, 1.34	0.001

*CI* confidence interval, *A&E* Accident and Emergency department, *IDACI* Income deprivation affecting children index, *IRR* Incident Rate Ratio, *LOS* Length of stay, *OR* Odds ratio, *ref* reference category

Model 1: Adjusted for admission hospital and quartile of IDACI; as defined in Tables 1 and 2

Model 2: Additionally adjusted for age (groups), gender, ethnicity (group) - as defined in Tables 1 and 2.

Model 3: Additionally adjusted for final diagnosis, medical or surgical specialty and number of hospital admissions during the study period

Model 4: Additionally adjusted for source of admission, weekend or week day admission, and season and time of admission - as defined in Tables 1 and 2)

**Table 7:** Model 4: Association of clinical, demographic and process factors with Prolonged length of stay

LOS:		>1.8 days (mean)				>3 days			>24 hours (median)		
Characteristic			OR	95% CI	p value	OR	95% CI	p value	β co-e	95% CI	p value
Demographic	Age group (years)	1 and Under	1.00	-	-	1.00	-	-	ref	-	-
		2 to 5	0.71	0.59, 0.86	0.001	0.62	0.49, 0.79	<0.001	−0.34	−0.56, −0.11	0.003
		6 to 10	0.72	0.54, 0.95	0.019	0.71	0.51, 0.99	0.045	−0.3	−0.61, 0.008	0.056
		11 to 16	0.88	0.64, 1.20	0.421	0.58	0.39, 0.86	0.007	−0.31	−0.65, 0.03	0.077
	Distance (overall quartiles)*	1st (closest)	1.00	-	-	1.00					
		2nd	0.70	0.85, 1.31	0.002	0.77	0.58, 1.01	0.059	−0.23	−0.50, 0.03	0.086
		3rd	0.82	0.56, 0.88	0.094	0.78	0.59, 1.02	0.073	−0.31	−0.57, −0.05	0.021
		4th (furthest away)	0.95	0.75, 1.21	0.707	1.03	0.77, 1.37	0.844	−0.15	−0.43, 0.12	0.271
Clinical	Specialty	Medical	1.00	-	-	1.00	-	-	ref	-	-
		Surgical	0.68	0.48, 0.96	0.030	0.75	0.48, 1.17	0.206	−0.32	−0.68, 0.05	0.088
	Diagnosis	allergy	1.00	-	-	1.00	-	-	ref	-	-
		cardiac	1.21	0.29, 5.10	0.796	0.90	0.18, 4.47	0.902	0.84	−0.86, 2.53	0.332
		endocrine	1.25	0.34, 4.70	0.736	1.30	0.29, 5.73	0.729	0.79	−0.77, 2.35	0.321
		gastro	0.84	0.26, 2.71	0.765	0.55	0.14, 2.10	0.382	0.02	−1.35, 1.40	0.972
		haemoncology	1.81	0.54, 6.09	0.341	1.48	0.37, 5.87	0.576	0.8	−0.62, 2.23	0.270
		infection	1.16	0.36, 3.71	0.807	0.61	0.16, 2.32	0.471	0.23	−1.14, 1.60	0.742
		MSK	0.69	0.18, 2.61	0.581	0.38	0.08, 1.93	0.245	0.19	−1.35, 1.73	0.809
		neurology	0.75	0.23, 2.46	0.631	0.61	0.16, 2.39	0.481	0.23	−1.16, 1.63	0.742
		other	0.92	0.26, 3.26	0.891	0.45	0.10, 1.98	0.292	−0.02	−1.51, 1.46	0.976
		psychosocial	0.41	0.10, 1.62	0.202	0.27	0.05, 1.60	0.150	−0.24	−1.79, 1.32	0.767
		respiratory	1.09	0.34, 3.50	0.885	0.60	0.16, 2.27	0.450	0.27	−1.10, 1.64	0.700
		surgical GIT	4.76	1.21, 18.70	0.026	1.88	0.43, 8.29	0.405	1.09	−0.48, 2.66	0.174
		trauma	0.46	0.14, 1.52	0.205	0.28	0.07, 1.13	0.073	0.14	−1.25, 1.53	0.845
		urogenital	1.32	0.39, 4.44	0.652	0.72	0.18, 2.89	0.647	0.26	−1.16, 1.69	0.720
		Maxfax/Dental/ENT	0.97	0.27, 3.53	0.960	0.38	0.08, 1.75	0.212	0.37	−1.10, 1.84	0.621
		congenital	1.20	0.14, 10.06	0.863	1.63	0.18, 14.64	0.662	0.18	−2.12, 2.48	0.876
		Dermatology	1.55	0.44, 0.96	0.499	0.80	0.19, 3.42	0.769	0.24	−1.26, 1.73	0.756
	Admissions per year	1	1.00	-	-	1.00	-	-			
		2	1.01	0.81, 1.25	0.950	1.18	0.91, 1.53	0.217	0.25	0.002, 0.50	0.048
		3	1.50	1.07, 2.11	0.020	1.32	0.89, 1.96	0.166	0.24	−0.16, 0.63	0.237
		4 or more	1.21	0.89, 1.64	0.228	1.90	1.36, 2.66	<0.001	0.24	−0.12, 0.59	0.194
Process	Time of admission	9.00 to 16.59	1.00	-	-	1.00	-	-	ref	-	-
		17.00 to 21.59	1.19	0.97, 1.46	0.087	1.32	1.04, 1.68	0.023	0.18	−0.06, 0.41	0.151
		22.00 to 08.59	0.61	0.51, 0.75	<0.001	0.81	0.64, 1.03	0.084	−0.46	−0.68, −0.24	<0.001
	Day of admission	weekday	1.00	-	-	1.00	-	-	ref	-	-
		weekend	0.76	0.60, 0.97	0.027	0.72	0.53, 0.97	0.033	−0.17	−0.44, 0.10	0.209
	Source of admission	elective	1.00	-	-	1.00	-	-	ref	-	-
		emergency via ED	4.59	2.80, 7.51	<0.001	2.14	1.97, 3.81	0.010	0.53	0.10, 0.97	0.017
		emergency via other	2.34	1.38, 3.97	0.002	1.72	0.93, 3.18	0.084	0.19	−0.30, 0.67	0.452
		other	8.68	4.16, 18.14	<0.001	6.96	3.18, 15.23	<0.001	3.01	2.24, 3.79	<0.001
	Season of admission	spring	1.00	-	-	1.00	-	-	ref	-	-
		summer	0.83	0.66, 10.4	0.113	0.92	0.70, 1.21	0.541	−0.36	−0.62, −0.11	0.006
		autumn	1.06	0.85, 1.32	0.607	1.22	0.94, 1.60	0.134	−0.02	−0.27, 0.24	0.887
		winter	0.87	0.69, 1.09	0.216	0.97	0.74, 1.28	0.836	−0.18	−0.44, 0.08	0.167

*β co-e* β co-efficient, *CI* confidence interval, *ED* Emergency department, *ENT* Ears Nose and Throat, *GIT* Gastrointestinal, *IDACI* Income deprivation affecting children index, *LOS* Length of stay, *MaxFax* Maxillofacial, *OR* Odds ratio, *ref* reference category

Model 4: Adjusted for age (groups), gender admission hospital, quartile of IDACI; ethnicity (group), final diagnosis, medical or surgical specialty, number of hospital admissions during the study period, source of admission, weekend or week day admission, season of admission and time of admission (as defined in Tables 1 and 2)

## Abbreviations

A&E: Accident and Emergency Department
CI: Confidence interval
HES: Hospital Episode Statistics
IDACI: Income Deprivation Affecting Children Index
IMD: Index of Multiple Deprivation
IQR: Interquartile range
IRR: Incident Rate Ratios
LOS: Length of stay
LSOA: Lower super output area
NHS: National Health Service
OR: Odds Ratio
PCT: Primary care trust
SD: Standard deviation
SEP: Socio-economic status
UK: United Kingdom
UKP: United Kingdom Pound
US: United States.
